# Meeting Report for Mobile DNA 2010

**DOI:** 10.1186/1759-8753-1-20

**Published:** 2010-08-24

**Authors:** George Chaconas, Nancy Craig, M Joan Curcio, Prescott Deininger, Cedric Feschotte, Henry Levin, Phoebe A Rice, Daniel F Voytas

**Affiliations:** 1 Department of Biochemistry and Molecular Biology, University of Calgary, Calgary, Alberta, T2N 4N1, Canada; 2 Department of Microbiology and Infectious Diseases, University of Calgary, Calgary, Alberta, T2N 4N1, Canada; 3 Department of Molecular Biology and Genetics, The Johns Hopkins University, Baltimore, MD 21205, USA; 4 Howard Hughes Medical Institute, 4000 Jones Bridge Road, Chevy Chase, MD 20815-6789, USA; 5 The Wadsworth Center, Center for Medical Sciences, Albany, NY 12201, USA; 6 Tulane Cancer Center, Tulane University, New Orleans, LA 70112, USA; 7 Department of Biology, University of Texas at Arlington, Arlington, TX 76019, USA; 8 Eunice Kennedy Shriver National Institute of Child Health and Human Development, Bethesda, MD 20892, USA; 9 Department of Biochemistry and Molecular Biology, The University of Chicago, Chicago IL 60637, USA; 10 Department of Genetics, Cell Biology & Development, The University of Minnesota, Minneapolis, MN 55455, USA

## Abstract

An international conference on mobile DNA was held 24-28 April 2010 in Montreal, Canada. Sponsored by the American Society for Microbiology, the conference's goal was to bring together researchers from around the world who study transposition in diverse organisms using multiple experimental approaches. The meeting drew over 190 attendees and most contributed through poster presentations, invited talks and short talks selected from poster abstracts. The talks were organized into eight scientific sessions, which ranged in topic from the evolutionary dynamics of mobile genetic elements to transposition reaction mechanisms. Here we present highlights from the platform sessions with a focus on talks presented by the invited speakers.

## Keynote address

The meeting was launched with a Keynote Address by Frederic Bushman (University of Pennsylvania, USA). Bushman summarized the extensive work his laboratory has conducted over the past several years to better understand mechanisms of retroviral integration. The Bushman laboratory pioneered the use of high throughput sequencing methods to map large numbers of retrovirus insertion sites. Bushman described how this approach revealed a preference for HIV to integrate into transcription units and the features of transcriptionally active chromatin that characterize these preferred sites. The relevance of this work was brought home when Bushman discussed integration patterns in the genomes of patients who received retroviral gene therapy. A better mechanistic understanding of how integration sites are selected should lead to better therapeutic approaches and limit the mutagenic outcome that caused leukemia in some of these patients.

## Session 1: genome evolution

Over the past several years, mobile DNA has progressively moved from second fiddle to centre stage in the field of genome evolution. The 'genome evolution' session highlighted the intricate and intertwined evolutionary trajectories of parasitic elements and their host genomes. Presenters and audience alike were amazed by the omnipresence and bewildering diversity of mobile DNA as they swung from one branch of the tree of life to another (including plants, primates, fruit flies, fungi, bacteria and a grab bag of protists).

Although viruses are found virtually everywhere on the planet, it is in the oceans that their abundance and extraordinary diversity is the most impressive, as Curtis Suttle (University of British Colombia, Canada) explained. He argued that viruses should be viewed both as an essential component of the ecosystem and as a threat to cellular organisms.

A flurry of talks by Mark Batzer (Louisiana State University, USA), Brandon Gaut (University of California, Irvine, USA), Pierre Capy (CNRS, Gif-sur-Yvette, France) and Richard Cordaux (CNRS, Poitiers, France) presented the results of empirical and theoretical studies, showing how adaptive processes (for example, epigenetic defense mechanisms) as well as non-adaptive processes (for example, population size) and life history traits of the host species (for example, the breeding system and endosymbiosis) have helped to shape the diverse genome landscapes adorned by transposons.

Another group of presentations focused on the involvement of mobile elements in the function of the genome. Josefa Gonzalez (Stanford University, USA) drew on the power of *Drosophila *population genomics to uncover a set of rare transposon insertions likely to be involved in the flies' adaptation to temperate climates. Another approach presented by Cédric Feschotte (University of Texas, Arlington, USA), which relies on multi-species genome alignments to demarcate evolutionarily conserved and likely functional elements, revealed thousands of primate-specific conserved non-coding sequences derived from transposable elements. Another mode of transposon domestication, whereby the transposition enzymes themselves are co-opted for host genome function, was illustrated by Irina Arkhipova (Marine Biological Laboratory, USA). She discovered an intriguing group of reverse transcriptases in fungi that apparently serve an, as yet, uncharacterized cellular function. Brian Higgins (Princeton University, USA), provided evidence for the involvement of transposases in programmed genome rearrangement in the ciliate *Oxytricha*.

## Session 2: genome diversification

The genome diversification session contained an eclectic collection of talks on widely differing systems and organisms, unified by a common theme. Marjorie Oettinger (Massachusetts General Hospital, USA) discussed V(D)J recombination, the DNA rearrangement process that generates a highly assorted collection of immunoglobulin molecules. She reported data on a new regulatory mechanism for V(D)J shuffling. The C-terminus of the recombination-activating gene 2 (RAG2) protein (one of the recombinase subunits) preferentially recognizes histone H3 that is dimethylated at Arg2 and trimethylated at Lys4, and methylation increases V(D)J recombination. Another layer of regulation may be contributed by chromatin accessibility modulated by the DNA binding protein CCCTC-binding factor, whose expression is regulated by an extracellular signal.

Genome diversification in ciliated protozoans was also a focal point of the session, with talks by Mireille Betermier on *Paramecium *(CNRS, Gif-sur-Yvette, France) and Alexander Vogt on *Tetrahymena *(Austrian Academy of Science). During the sexual development of these organisms a new macronucleus is derived from the germline which involves a process referred to as DNA elimination. During this process a large number of internal eliminated sequences (IESs) are removed. Both groups have shown that this process is driven by a domesticated piggyBac transposase in *Paramecium *and *Tetrahymena*, providing yet another example of a domesticated transposase with an important function. The domesticated transposase localizes to the new developing macronucleus in both organisms. The CNRS group has also highlighted a role for DNA ligase IV/XRCC in completing the DNA elimination reaction.

Other examples of a genome diversification process are the DNA rearrangements underlying changes in surface proteins (antigenic variation) by which pathogens escape immunosurveillance in the host. Nina Papavasiliou (Rockefeller University, USA) reported that induction of a double-stranded break (DSB) contiguous to the expressed variant surface glycoprotein (VSG) gene of *Trypanosoma brucei *results in a 200-fold increase in VSG recombination. Moreover, naturally occurring DSBs were detected upstream of the expression locus, suggesting that DSBs may be the trigger that initiates the DNA rearrangements underlying antigenic switching. The mechanism of DNA switching at the *vlsE *locus in *Borrelia burgdorferi *is being studied in the laboratory of George Chaconas (University of Calgary, Canada). He reported that, out of the 14 genes identified in antigenic switching in *Neisseria*, only the *ruvAB *function is required in *B. burgdorferi. *The lack of a requirement for *recA *and other recombination/repair functions may be explained by the observed ability of DNA sequences in the *vls *locus to promote synapsis and template strand switching by DNA polymerase to generate recombinant DNA molecules. These findings suggest an unusual DNA-driven mechanism for antigenic switching that reduces protein involvement in the recombination process at the antigenic variation locus in this organism.

A very unusual mechanism of genome diversification was discussed by Jeff F. Miller (University of California, Los Angeles, USA). Diversity-generating retroelements (DGRs) are found in bacteria and phage, including human pathogens. These elements direct nucleotide substitutions (A to any base) within specific regions in protein coding sequences in target genes through a mutagenic homing pathway. The results are a constellation of diversified proteins with altered properties. Such sequence scrambling can offer a selective advantage for protein optimization through diversification, particularly in surface receptor proteins. The non-proliferative copy-and-replace mechanism involves integration of diversified complimentary DNA (cDNA) copies generated by the DGR-encoded reverse transcriptase.

## Session 3: DNA transposons

The very interesting talks in the 'DNA transposons' session ranged from an *in vitro *high-resolution crystallographic dissection of transposase architecture to an *in vivo *analysis of the roles of host proteins in transposition. They provided answers to some long-standing issues in the field and also provided exciting new topics to be explored.

Fred Dyda (National Institutes of Health, USA) discussed the known structures of DNA transposases and retroviral integrases of the DDE type. He emphasized that, while we do know the structure of several such enzymes, many more transposable elements have been bioinformatically described for which there has been no experimental work. Will all these other elements fit the DDE paradigm and transpose using similar chemical steps? Julia Richardson (University of Edinburgh, UK) presented her structure of the *Mos1 *transposase bound to DNA. Notably, the configuration of the *Mos1 *transposon ends is quite distinct from that of *Tn5*, the only other DNA-transposase co-crystal. In *Mos1*, the two transposon ends are positioned in parallel in the active sites of the transposase dimer as opposed to the anti-parallel alignment in *Tn5*.

Several talks focused on how cleavage at the transposon ends occurs. Corentin Claeys Bouuaert (University of Nottingham, UK) reported experiments also using *Mos1 *that support the view that the active transposase form is indeed a dimer but that a single protomer bound to each end carries out a double strand break - that is, cleaves both the 5' and 3' strands at each end. However, it remains to be determined how a single protomer can promote cleavage of both DNA strands. Rasika Harshey (University of Texas, USA) provided a key insight into a long-standing issue in Mu transposition of how donor site DNA that contains the 5' ends of the transposon is cleaved upon integration into the bacterial chromosome to form the Mu lysogen. She has identified a 5' end endonuclease activity in the Mu transposase that performs the 3' end cleavage and target-joining steps.

Two other talks dealt with how transposon target sites are found. Joe Peters (Cornell University, USA) discussed his findings that the *Tn7 *TnsE protein interacts with the bacterial processivity factor and is thus recruited preferentially to replicating DNA. Nancy Craig (Johns Hopkins University, USA) presented work demonstrating that, in a heterologous *Saccharomyces cerevisiae *system, the favoured chromosomal targets of the insect *hAT *element *Hermes *are nucleosome-free regions.

## Session 4: non-long terminal repeat (LTR) retrotransposons and group II introns

The session on non-LTR retrotransposons and group II introns illustrated both the continued activity of these elements in altering the human genome and the remarkable diversity of regulatory mechanisms of the various non-LTR elements. Talks by Haig Kazazian (University of Pennsylvania, USA) and John Moran (University of Michigan, USA) described new approaches to detect the remarkable level of ongoing L1 activity in the human genome. The Kazazian talk utilized a polymerase chain reaction (PCR)-based approach combined with next generation sequencing to look at L1 diversity in 25 different human genomes. He found that there were typically about 285 L1 inserts which were polymorphic between any two different individuals and approximately 10,000 such polymorphisms among the 25 individuals surveyed.

The Moran study utilized a paired-end sequencing procedure on fosmids from different individuals that allowed identification of full-length and, therefore, potentially active elements in six geographically distinct individuals. This approach avoids the use of PCR which aided in testing the retrotransposition potential of these elements, and they found that highly active elements are much more common in the genome than previously predicted.

The L1 talks were rounded out by Elena Khazina (Max-Planck-Institute for Developmental Biology, Tuebingen, Germany), who presented an elegant model of L1 open reading frame (ORF)1p function based on crystal structure determination at the 1.4 angstrom level. She found a strikingly flexible structure between the required RNA recognition motif (RRM) and C-terminal (CTD) domain that suggests a need for a dynamic structure in functional ORF1p. Tom Eickbush (University of Rochester, USA) pushed his studies on the target-primed reverse transcription of insect R2 elements to a new level by demonstrating that their processing from the ribosomal transcripts in which they are initially made is the result of a conserved ribozyme domain that specifically cleaves at the 5' end of the functional R2 RNA.

There were two talks on SINEs from Prescott Deininger (Tulane Cancer Center, USA) and Jean-Marc Deragon (Université de Perpignan, France). Deininger summarized the structural features of human Alu elements that combine to silence all but the most recent Alu inserts. Variations in the A-tail led to the most rapid decline of activity with damage to the 3' end of the A-tail being most disruptive. These rules helped identify the most likely source element for an Alu insertion causing cystic fibrosis. Deragon described more diverse groups of short interspersed nuclear elements (SINEs) found in maize with a particular emphasis on SINE families that end in runs of T residues rather than As. The polythymine (polyT) end on these element families is not consistent with the classical target primed reverse transcription (TPRT) mechanism and suggests the need for an as yet unknown variation on the normal priming mechanism.

Marlene Belfort (Wadsworth Center, USA) spoke about group II introns, which are self-splicing RNAs found principally in prokaryotes and archaea. Group II introns are hypothesized to be precursors to eukaryotic spliceosomal introns. However, they are not found in eukaryotic nuclear DNA. By introducing a bacterial group II intron into the yeast genome, Belfort's group found that the intron is spliced effectively but the RNA is subject to nonsense-mediated mRNA decay and is poorly translated. This suggests a hostile eukaryotic genome may have promoted intron loss and the evolution of a protein-dependent splicing apparatus.

## Session 5: host-element interactions

The talks in the 'host-element interactions' session revealed the ever-expanding diversity of mechanisms by which host-element crosstalk reflects developmental changes in the organism and influences genome structure. Each talk focused on different host-element relationships, ranging from the highly domesticated a3 DNA transposon of the yeast, *Kluyveromyces lactis *to the exogenous HIV retrovirus that infects human cells.

Stefan Åström (Stolkhom University, Sweden) presented evidence that the *K. lactis *a3 mating-type (*MAT*) gene is a transposon that is excised from the chromosome as a circular DNA molecule. The α3-encoded protein contains a DDE motif that is conserved among transposases and is essential for the excision event and for mating type switching. Mobilization of the α3 transposon results in a chromosome break. Formation of the chromosome break induces the introduction of **a**-cell-specific information from another chromosomal locus, completing the switch from *MAT*α to *MAT*a. Excision circle formation, and therefore mating-type switching, requires the host Mts1 protein which, in turn, is regulated by the nutrient-sensing RAS/cyclic adenosine monophosphate (cAMP) pathway, thereby linking this transposon-mediated mating type switch to nutrient availability.

By comparison to the a3 transposon, the Ty1 retrotransposon of *S. cerevisiae *is not as domesticated. However, its mobility is regulated by environmental stresses through host cell signalling pathways and it facilitates chromosomal rearrangements, retrogene formation and telomere elongation in the absence of telomerase. Therefore, it is not surprising that Ty1 retrotransposition relies heavily on the host to carry out basic replication steps, such as Ty1 RNA packaging into virus-like particles. Joan Curcio (Wadsworth Center, USA) reported that packaging of Ty1 RNA requires the host 5' to 3' mRNA degradation pathways. Ty1 RNA is sequestered from translation in high molecular weight particles, and Ty1 Gag localizes to mRNA processing bodies, where the 5' to 3' mRNA degradation machinery is concentrated. Curcio speculated that Ty1 RNA packaging might be associated with the host mRNA degradation machinery to exclude cellular mRNAs from Ty1 virus-like particles.

Transposable element activity is frequently held in check by the activity of a nuclear immune system that silences potentially dangerous repetitive DNA. Damon Lisch (University of California, Berkeley, USA) talked about regulated expression of the *MuDR *elements of maize. He revealed a surprising loss of epigenetic control of *MuDR *expression in leaves that are undergoing a transition from juvenile to adult. Lisch speculates that there is a close relationship between the transition to reproductive maturity and mechanisms for transposon recognition and silencing.

How does the intimate host-element relationship begin? Sarah Schaack (University of Texas, Arlington, USA) presented recent findings that address this question. Transposable element families in a blood-sucking triatomine bug are almost identical to those found in the genomes of a variety of phylogenetically diverse animals that are related by their geographic distribution and by being hosts to the triatomine bug. The findings by Schaack and colleagues highlight the central role that parasites may play in introducing transposable elements (TEs) from one animal species to another by horizontal transfer. The subsequent expansion of horizontally transferred TEs is likely to impact the structure of the host genome.

Alan Engelman (Dana-Farber Cancer Institute, USA) rounded off the session by discussing the idea of exploiting the host-element relationship. The lentiviral integrase binding protein, lens epithelium-derived growth factor (LEDGF) is involved in targeting HIV to active genes during integration. Altering the chromatin binding capacity of recombinant LEDGF fusion proteins redirected HIV to novel integration sites, suggesting that LEDGF fusions could one day help steer lentiviral gene therapy vectors to benign regions of the human genome. Engelman also presented a new model for the active HIV intasome nucleoprotein complex based on the recent foamy virus integrase-DNA crystal structure, which highlighted novel HIV integrase-DNA contacts.

## Session 6: LTR retrotransposons

The 'LTR retrotransposon' session began with a talk by Dan Voytas (University of Minnesota, USA) who described work from his laboratory on the LTR-retrotransposon Ty5 and its behaviour in *S. cerevisiae*. Mapping the positions of individual integration sites revealed that Ty5 integrates specifically into regions of heterochromatin such as the mating type locus and telomeres. In order to get a comprehensive view of insertion sites throughout the genome, Voytas used ligation-mediated PCR and 454 pyrosequencing. This approach provided thousands of insertion sites and was able to show that a full 5% of the insertions occurred in euchromatin. Interestingly, the insertions in euchromatin clustered upstream of ORFs. Additional analysis revealed that Ty5 integration avoided ORFs and nucleosomes but showed an association with origins of replication.

DNA methylation in mammalian cells is known to repress the activity of transposable elements. Tetsuji Kakutani (National Institute of Genetics, Japan) described his study of DNA methylation in *Arabidopsis thaliana*. The protein DDM1 is responsible for methylating transposon sequences. Strains lacking DDM1 exhibited very high levels of transposition of the elements ATGP3, ATCOPIA13, ATCOPIA21 and ATCOPIA93. Kakutani also described wild isolates of plants that contained recent bursts of transposition of ATCOPIA93 all of which were found in centromeres. The patterns of transposition caused by the mutation of *ddm1 *were similar to those in wild plants suggesting that the DDM1 system serves as an important model for the mobility of transposons in *A. thaliana*.

The LTR-retrotransposon Tf1 of *Schizosaccharomyces pombe *integrates with a strong preference for the promoters of pol II-transcribed genes. Henry Levin (National Institutes of Health, USA) described the use of pyrosequencing to identify large numbers of Tf1 insertions. These data provided a highly reproducible measure of integration levels within each promoter of the genome. Interestingly, the bulk of Tf1 integration occurred within just 20% of all promoters. Levin also described the transposition activity of Hermes, a DNA 'cut and paste' transposon from the housefly. Hermes exhibits high levels of transposition in *S. pombe *and 33% of the inserts occurred within ORFs. With this system, Levin used pyrosequencing of integration in a haploid to map the essential genes of *S. pombe. *This approach represents a novel application of pyrosequencing for mapping essential sequences throughout the genome.

## Session 7: site-specific recombinases and their relatives

Classic site-specific recombinases from the serine family were featured in two talks. Both of these recombinases are regulated by substrate topology and are activated in the context of a larger complex that promotes a dimer-tetramer transition. Reid Johnson (University of California, Los Angeles, USA) detailed extensive cross-linking of Hin invertase and its host cofactor Fis, leading to a well-supported model for the full synaptic complex. Marshall Stark (University of Glasgow, Scotland) described an extensive analysis of the conserved residues found in the catalytic site of Tn3 resolvase and the very surprising result that only the very C-terminal portion of the catalytic domain is required for synaptic tetramer formation.

Tyrosine recombinases comprise the other 'classic' family of site-specific recombinases. CTnDOT, a conjugative transposon integrase, is an unusual example that was described by Jeff Gardner (University of Illinois, USA). Unlike other family members, this integrase is not deterred by mismatches within its crossover site. Sequence alignments also imply that it is missing a critical catalytic residue. However, structural modelling and biochemical experiments show that it may be replaced by another residue from a different portion of the primary sequence that could occupy the same position in the tertiary structure.

Three talks focused on the IS200/605 family of 'Y1' transposases. These enzymes are a branch of the His-hydrophobic-His motif (HUH) superfamily of nucleases and are remarkable among transposases for acting on single-stranded DNA substrates and recognizing a short target sequence through DNA-DNA contacts. Mick Chandler (CNRS, Toulouse, France) described how these elements target replication forks while Laure Lavatine from the Chandler laboratory presented data showing that IS608 transposase is targeted to stalled replication forks *in vivo*, perhaps through the influence of host factors. Jaroslav Nunvar (Charles University, Prague, Czech Republic) described the genomic association of a subfamily of these transposases with bacterial repetitive extragenic palindromic (REP) elements, suggesting that REP elements may be mobilized by Y1 transposases. Finally, Phoebe Rice (University of Chicago, USA) presented a crystal structure of an active complex of bacteriophage Mu transposase, a DDE-family enzyme, bound to both phage ends and target DNA.

## Session 8: biological impacts of transposition

The conference was capped with a session addressing biological impacts of transposition and included talks that provided a new understanding of key transposition mechanisms as well as additional insights into how mobile elements shape the biology of their hosts.

Peter Cherepanov (Imperial College, UK) reported on his recent and much anticipated structure of the complete foamy virus integrase bound to DNA. Cherepanov observed extensive protein-DNA and protein-protein interactions among the three structural domains of integrase. Binding of strand-transfer inhibitors displaced viral DNA from the active site and the extension of these studies should aid in the development of new antiretroviral drugs. Further, this work allows the structural modelling of other retroviral integrases, including HIV. Mechanisms of DNA mobility were also addressed by Alan Lambowitz (University of Texas, Austin, USA). The Lambowitz group studies mobile group II introns, and work was presented describing the mechanisms that contribute to the proliferation of group II introns in thermophilic cyanobacteria. Interestingly, group II introns from thermophiles rely on elevated temperatures to help promote DNA strand separation, making it possible for them to utilize a larger number of DNA target sites through base pairing with the intron RNA. Jef Boeke (Johns Hopkins University School of Medicine, USA) moved one step away from the transposition reaction to assess effects of chromatin on retrotransposon target site choice. Boeke's element of study was Ty1 from *S. cerevisiae*, which prefers to integrate upstream of genes transcribed by RNA polymerase III. Using high throughput DNA sequencing to analyze thousands of Ty1 integration events, Boeke provided evidence that Ty1 integrates preferentially into nucleosomes upstream of preferred targets. A clear integration hotspot was observed in the nucleosome-bound DNA, suggesting histone modifications may dictate this integration bias.

Rob Martienssen (Cold Spring Harbor Laboratory, USA) moved the discussion further into the realm of host/element interactions. The Martienssen group has shown that otherwise silent transposable elements become activated during *Arabidopsis *pollen and egg development. Activation occurs in somatic companion cells that do not provide DNA to the fertilized zygote. Small RNAs corresponding to transposable elements in these somatic cells are thought to reprogram heterochromatin in the germline to silence mobile elements and preserve genome integrity in the next generation. The theme of host-element interactions was further developed by Harmit Malik (Frederick Hutchinson Cancer Institute, USA). Malik described the new field of paleovirology - the study of ancient, extinct viruses and how they impact host genome evolution. The most obvious paleoviruses are the endogenous retroviruses that riddle most vertebrate genomes. Malik suggests that other paleovirus infections influenced the evolution of host defense mechanisms and thereby the repertoire of defense strategies available to combat emerging viruses.

## St Malo 2012

The tradition of a large international meeting on 'mobile DNA' will continue. In the spring of 2012, the International Congress on Transposable Elements will be held in St Malo, France. Stay tuned for more details!

## Abbreviations

A: adenine; DDM1: deficient in DNA methylation 1 protein; DGR: diversity-generating retro element; DSB: double-stranded break; LEDGF: lens epithelium-derived growth factor; LTR: long terminal repeat; MAT: mating-type; mRNA: messenger RNA; ORF: open reading frame; PCR: polymerase chain reaction; REP element: repetitive extragenic palindromic element; SINE: short interspersed nuclear elements; TE: transposable element; VSG: variant surface glycoprotein gene.

## Competing interests

The authors declare that they have no competing interests.

